# What works for wellbeing in culture and sport? Report of a DELPHI process to support coproduction and establish principles and parameters of an evidence review

**DOI:** 10.1177/1757913916674038

**Published:** 2016-10-28

**Authors:** Norma Daykin, Louise Mansfield, Annette Payne, Tess Kay, Catherine Meads, Giorgia D’Innocenzo, Adele Burnett, Paul Dolan, Guy Julier, Louise Longworth, Alan Tomlinson, Stefano Testoni, Christina Victor

**Affiliations:** Professor, Centre for the Arts as Wellbeing, The University of Winchester, Sparkford Road, Winchester SO22 4NR, UK; Brunel University London, Uxbridge, UK; Brunel University London, Uxbridge, UK; Professor, Brunel University London, Uxbridge, UK; Westbrook Centre, RAND Corporation Europe, Cambridge, UK; Brunel University London, Uxbridge, UK; Brunel University London, Uxbridge, UK; Professor, London School of Economics, London, UK; Professor, University of Brighton, Brighton, UK; Brunel University London, Uxbridge, UK; Professor, University of Brighton, Brighton, UK; London School of Economics, London, UK; Professor, Brunel University London, Uxbridge, UK

**Keywords:** culture, sport, wellbeing, evidence review, coproduction, DELPHI

## Abstract

**Aims:**

There is a growing recognition of the ways in which culture and sport can contribute to wellbeing. A strong evidence base is needed to support innovative service development and a 3-year research programme is being undertaken to capture best evidence of wellbeing impacts and outcomes of cultural and sporting activities in order to inform UK policy and practice. This article provides an overview of methods and findings from an initial coproduction process with key stakeholders that sought to explore and agree principles and parameters of the evidence review for culture, sport and wellbeing (CSW).

**Methods:**

A two-stage DELPHI process was conducted with a purposeful sample of 57 stakeholders between August and December 2015. Participants were drawn from a range of culture and sport organisations and included commissioners and managers, policy makers, representatives of service delivery organisations (SDOs) and scholars. The DELPHI 1 questionnaire was developed from extensive consultation in July and August 2015. It explored definitions of wellbeing, the role of evidence, quality assessment, and the culture and sport populations, settings and interventions that are most likely to deliver wellbeing outcomes. Following further consultation, the results, presented as a series of ranked statements, were sent back to participants (DELPHI 2), which allowed them to reflect on and, if they wished, express agreement or disagreement with the emerging consensus.

**Results:**

A total of 40 stakeholders (70.02%) responded to the DELPHI questionnaires. DELPHI 1 mapped areas of agreement and disagreement, confirmed in DELPHI 2. The exercise drew together the key priorities for the CSW evidence review.

**Conclusion:**

The DELPHI process, in combination with face-to-face deliberation, enabled stakeholders to engage in complex discussion and express nuanced priorities while also allowing the group to come to an overall consensus and agree outcomes. The results will inform the CSW evidence review programme until its completion in March 2018.

## Background

There is a growing recognition of the ways in which culture and sport can contribute to wellbeing. Developments in policy including local commissioning of needs-based health and social care have created new opportunities for culture and sport activities to be integrated into service delivery.^1^ However, a strong evidence base is needed to support innovations of this kind. The UK What Works Wellbeing Centre has commissioned a 3-year programme of research synthesis and secondary data analysis across three areas: work and learning, community wellbeing, and culture and sport. The evidence reviews are funded by the Economic and Social Research Council from June 2015 to May 2018. This article reports the initial consultation process for the Culture, Sport and Wellbeing (CSW) review. The review is a collaboration between four UK universities and seeks to determine wellbeing impacts in culture and sport practices in diverse communities and contexts, and to establish how evidence can be used effectively to inform policy and practice decisions. This article briefly introduces the policy context relating to culture and sport as well as concepts of wellbeing relevant to the sectors. We then present an overview of the methods and findings from a two-stage DELPHI process with key stakeholders that sought to explore and agree principles and parameters of the CSW evidence review programme.

The importance of measuring and understanding subjective wellbeing (SWB) is becoming established in policy and decision-making in the United Kingdom.^2–4^ Recent developments have stimulated policy-related recognition of the importance of measuring and understanding SWB.^5^ Since 2011, the Office of National Statistics (ONS) has assessed ‘personal well-being’ or ‘subjective wellbeing’ using four dimensions: satisfaction with life, worthwhileness, happiness and anxiety (the ONS4). Recognition of the significance of leisure practices for individual and collective wellbeing was formalised through the inclusion of measures of cultural and sporting engagement in SWB data collection by the ONS from 2013.

Scholars have long-recognised the connections between culture and wellbeing, though in varied terminology. The 1930s historians and economists saw spheres of arts and leisure/amusement as antidotes to Marxist notions of alienated labour or routes towards a well-lived life.^6,7^ In the 1970s, leisure providers saw cultural, sports and community interventions as a means of enhancing quality of life.^8,9^

Worldwide evidence on the drivers, impact and value of engagement in culture and sport is available,^10^ with positive effects on wellbeing relationships reported for taking part in sports, arts, heritage, museums, libraries, and archive activities.^11^ Significant associations between wellbeing and engagement in cultural and sport practices have been calculated and a financial value placed on participation in the arts (£1,084/yr), libraries (£1,359/yr) and sports (£1,127/yr).12 Physical activity is reported to be associated with increased life satisfaction, one of the ONS4 dimensions of wellbeing, for both men and women.^13^

Within the culture sector, a growing body of research has examined the impacts of arts programmes on health and wellbeing in both clinical and community contexts.^14,15^ The relevance of arts to public health concerns has been recognised.^15,16^ As well as examining the impact of arts on individuals, research has linked cultural interventions with personal and collective wellbeing benefits for particular social groups, including improvements in public health,^17^ reduced inequalities,^18^ cultural value and social capital.^19^

While the evidence in support of positive wellbeing impacts of culture and sport is growing, there is still a lack of consensus in policy, academic and practice circles about how to conceptualise wellbeing and best methods for measuring wellbeing outcomes.^5,11,20–23^ Most recently, policy makers and academics have focused on conceptualising and measuring SWB; the feelings, experiences and sentiments arising from what people do and how they think.^20^ To date, SWB has been most often assessed by asking people to provide global and retrospective evaluations of their life and experiences (e.g. in national and international surveys). There is a distinction between evaluative and experiential measures of SWB, the former capturing how people think they feel overall, the latter identifying how people feel over time based on what they are doing in a particular setting. Evaluative approaches to SWB are useful to policy making but are likely to provide only partial representations of people’s wellbeing experiences over time and in different contexts. It has been found that cultural and physical activity–related activities (e.g. going to the theatre/museums, dancing, performing music and reading) are strongly associated with improved *experienced* happiness and reduced anxiety.^24^

In terms of evidence review work, there are ongoing questions about what criteria to use in judging the evidence for CSW, as well as concern about the influence of hierarchies of evidence drawn from medicine and health disciplines.^25^ Frameworks need to recognise the broad range of evidence types currently used to inform policy and practice decision-making.^26,27^ There is some reported ambivalence from commissioners, managers, deliverers and researchers working in sport and culture towards the imposition of evaluation models that privilege methodologies that may not be appropriate for these contexts.^1^

## Aims

The project seeks to work with stakeholders to develop a shared understanding of what works, and what does not work, in culture and sport in order to inform public policy and funding priorities as well as professional practice. The project commenced with a 6-month collaborative development phase, which included a two-stage DELPHI activity involving 57 participants from the UK culture and sport sectors, designed to establish agreed principles and parameters for the 3-year CSW evidence review programme (see Supplementary Material). The DELPHI approach offered a way to build consensus through an iterative, structured process that gives all participants an equal voice.

## Methods

A 6-month collaborative process including face-to-face workshops, interviews, observations and consultation with stakeholders informed a two-stage DELPHI process to establish priorities for the CSW evidence review. The DELPHI technique originated in the late 1940s as a method of aggregating the judgements of a number of individuals in order to improve the quality of decision-making.^28^ It involves sending questionnaires to participants, sometimes in more than one round, asking them to state their level of agreement with a series of statements. The technique can guide policy and service development, supporting priority-setting while allowing a wide range of people to contribute to decisions.^29^ People do not have to meet together in order to complete the DELPHI process, which makes it a relatively efficient and accessible methodology. However, the process and data are enriched by building on prior face-to-face discussions.^30^ This allows the questionnaires to include statements generated by participants and stakeholders rather than by researchers. Participants’ responses are often ranked using mean scores, producing a group judgement. The outcomes of a DELPHI process can be influenced by the methods for analysing the results, particularly by the decision where to place the threshold of consensus. This can be high or low depending on the aims of the particular exercise. If a high threshold is adopted, there will be fewer resulting statements, but these may be too bland to be useful.^30^ If a low threshold of consensus is adopted, the statements may mask important disagreements.

An initial questionnaire (DELPHI 1) was devised following consultation at a 2-day event at Brunel University London in July 2015 with a purposeful sample of 34 delegates drawn from national and regional policy, commissioning, research and service delivery organisations (SDOs) in culture and sport. The initial sample was identified by the research team, which includes culture and sport academics from four UK universities, who have been working and collaborating with a wide range of sector stakeholders for several years. Snowball sampling identified further respondents suggested by participants and through consultation with the national What Works delivery partners.

Telephone interviews were subsequently conducted with 20 stakeholders who could not attend the workshops. Participants discussed definitions of SWB, research challenges and priorities for the CSW evidence review. Data were audio recorded and transcribed and thematic analysis was undertaken using principles outlined by Braun and Clarke.^31^ The results were discussed with a sub-sample of professionals, practitioners and service users from the participating organisations at a series of dissemination events in December 2015, including sessions where researchers observed activities delivered by culture and sport SDOs and discussed evaluation issues with participants. Written informed consent was taken for all data capture activities. The project received ethics approval from the Brunel University London Ethics committee (reference CHLS-RE41-14).

**Figure 1.**
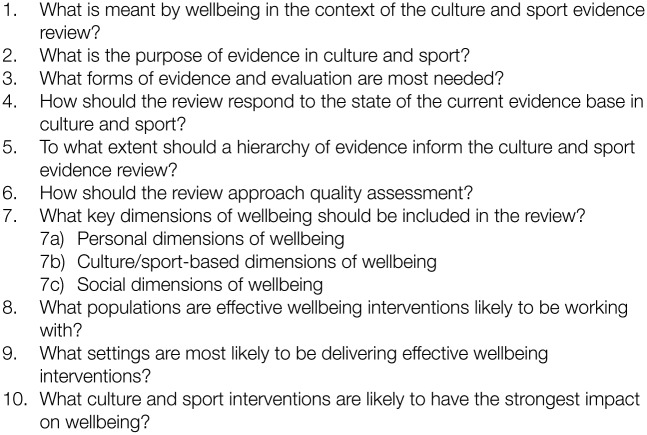
A total of 10 questions on principles and parameters of the CSW evidence review.

Thematic analysis^31^ of project data revealed four broad themes including definitions and dimensions of wellbeing, the purpose of evaluation, hierarchies of evidence, and culture, health and wellbeing populations, settings and interventions. These data will be reported on in full in a different paper. Here, we describe the next stage of the consultation process, which sought to establish the key principles and map the parameters of the review using the DELPHI method. The data were drawn together into 10 questions addressed at stakeholders (Figure 1). These were elaborated in a 10-item DELPHI 1 questionnaire (Supplementary Table 1).

For each question, respondents were asked to rate how much they agreed or disagreed with up to eight pre-set responding statements by rating each of them from 5 (*agree strongly*) through 3 (*neither agree nor disagree*) to 1 (*disagree strongly*). The statements drew directly on the interpretive process of the qualitative data and adopted the same language and phrasing used by participants during discussions and conversations in order to ensure a grounded approach to the consensus development process. Hence, the questionnaire design process was inductive: the questionnaire, while it covers many issues that one would expect to see in a discussion of evidence review for culture and sport, is specific to the project and not intended as a generally applicable tool.

The DELPHI 1 questionnaire was sent to 54 participants identified from the workshops including commissioners and managers (14), policy makers (14), scholars and fellows (13), and representatives of SDOs (13). Questionnaire data were entered into a spreadsheet and imported into statistical analysis software (IBM SPSS Statistics v. 20; IBM, Armonk, NY). As well as examining overall rankings, we compared the rankings for different sub-groups with the overall means in order to explore possible disagreements and nuanced standpoints of the different stakeholders. In view of the small numbers in the groups, we calculated *p*-values and significance using unpaired *t*-tests for this purpose. An analysis of variance (ANOVA) test compared the means of groupings with each other to identify similarities and differences in group preferences.

The results were shared with stakeholders in collaborative dissemination events before a second questionnaire was sent out to 57 people (the original DELPHI 1 sample plus three additional stakeholders who we identified during collaboration). In the DELPHI 2 questionnaire, the top five statements from DELPHI 1 were presented in rank order, with a rank of 1 indicating the most important statement, a rank of 2 indicating the second most important statement and so on. Out of a total of 69 statements, 9 were excluded at DELPHI 2; hence, this second questionnaire was not substantially different in scope from DELPHI 1. The purpose of DELPHI 2 was to offer participants the opportunity to mark any disagreements with the DELPHI 1 results. However, given that the DELPHI 1 results had been arrived at via a considerable consultation process we did not expect substantial disagreements at this stage.

Participants who disagreed with the order in which the statements were presented were able to assign new ranks; otherwise, they did not assign new ranks to the statements. It was made clear that answers that were left blank would be treated as agreeing with the initial rankings.

## Results

### DELPHI 1

The DELPHI 1 questionnaire was returned by 38 participants: commissioners and managers (9), policy makers (11), scholars and fellows (10), and representatives of SDOs (8) (Supplementary Table 1). In one case, three people from the same organisation completed a joint response, which was counted as a single response. The results (Supplementary Table 1) show the range of priorities and illustrate key issues that are pertinent to the review process, including definitions and dimensions of wellbeing, the role of evidence, approaches to quality assessment, and the populations and settings and interventions most likely to deliver wellbeing in culture and sport. The statements and rankings reflect the range of responses discussed during workshops and interviews.

As well as determining the overall rankings of statements, we examined variations in ratings between the groups using unpaired *t*-tests. We selected statements that were ranked first or second by sub-groups where these statements were ranked lower than fourth overall. There were five such statements (Supplementary Tables 2–5).

When asked ‘What is meant by wellbeing in the context of the culture and sport evidence review’, the SDO group gave second ranking to the statement ‘we need to adopt an inductive approach that assesses wellbeing in each specific context’ (mean = 1.5, standard deviation (SD) = 0.926). This statement was ranked fifth overall (mean = 2.32, SD = 0.989). The difference was significant (*p* = .0369). SDO rankings also differed from the overall rankings in their responses to the statement, ‘To what extent should a hierarchy of evidence inform the culture and sport evidence review?’ The statement ‘The review should include anecdotal evidence and testimonials’ was ranked first by SDOs (mean = 1.38, SD = 1.061) compared with fifth overall (mean = 2.58, SD = 1.030). This difference was very significant (*p* = .0047). There were non-significant (*p* = .5041) differences in rankings of the statement, ‘Evaluation should be embedded in practice and should not disrupt service delivery’. This was ranked second by SDOs (mean = 1.43, SD = 1.134) compared with fourth overall (mean = 1.73, SD = 1.071).

Supplementary Tables 3 and 4 show differences in preferences of commissioners and managers, scholars and policy makers, although none of these were significantly different. In response to the question ‘What forms of evaluation are most needed?’, commissioners and managers gave first ranking to the statement ‘Case studies and stories are needed in order to explain the wellbeing benefits of participation in culture and sport to participants and funders’ (mean = 1.56, SD = 1.333). This statement was ranked fifth overall (mean = 1.76, SD = 1.149).

When asked ‘what is the purpose of evidence in culture and sport?’, scholars gave second ranking to the statement ‘Evaluation should be as independent as possible’ (mean = 1.40, SD = 0.699), compared with fifth overall (mean = 1.87, SD = 1.07). Policy makers ranked this statement third (mean = 1.87, SD = 1.070). An ANOVA test revealed that overall, the groups voiced similar preferences with few significant differences. Hence, the scholars and policy makers did not have a significant difference of opinion from each other, or all the respondents taken together.

The analysis shows that there were just two statements in relation to which significant differences between groups were found, both of which related to SDOs. The statistics show that with these two exceptions all the groups are in agreement, giving very similar responses to the questions in DELPHI 1. This would suggest that the resulting consensus statement can enable a common strategy to be drawn up to assess the effect of policies and initiatives on wellbeing with which all the groups can agree.

### DELPHI 2

Two commissioners/managers who had not responded to DELPHI 1 completed DELPHI 2 (Supplementary Table 2), bringing the total of responses to 40 (70.02%). After two follow-up communications, eight respondents returned the questionnaire with the original rankings confirmed, or sent an email or spoke to a researcher on the phone confirming their agreement. A further six did not reply and, as they had been advised, their responses were logged as signalling agreement. A total of 26 respondents returned the questionnaire with minor changes, but these did not result in any changes to the overall rankings (Supplementary Tables 5–10). The final results show a strong pattern of agreement, which is perhaps unsurprising given that the DELPHI 2 questionnaire came at the end of a 6-month in-depth face-to-face and online consultation. To a degree, all the statements are important to stakeholders and have the potential to inform the evidence review. While it is not necessary for the purposes of this exercise to impose a rigid threshold of consensus, here we make a perhaps arbitrary distinction between statements that made it into the top three and those ranked fourth or fifth in order to highlight key priorities.

In response to the question ‘What is meant by wellbeing in the context of the culture and sport evidence review?’ (Supplementary Table 5), the highest ranked statement (mean = 1.5, SD = 1.109) was ‘we need a common definition of wellbeing on which to base evaluation and research’ followed by ‘We need to include a range of wellbeing dimensions into evidence reviews in addition to the ONS4’ (mean = 2.20, SD = 0.648) and ‘The ONS four dimensions of wellbeing (life satisfaction, worthwhileness, happiness and anxiety) are important to the sector’ (mean = 3.08, SD = 0.694). Lower priority was given to ‘We need to avoid reductionist, over-simplistic approaches in order to capture complex dimensions of wellbeing’ (mean = 3.73, SD = 0.960) and ‘We need to adopt an inductive approach that assesses wellbeing in each specific context’ (mean = 4.48, SD = 1.198).

Participants were asked to respond to the question, ‘what is the purpose of evidence in culture and sport?’ (Supplementary Table 6). The top three statements were the following: ‘Evidence is needed to secure and maintain funding for culture and sport’ (mean = 1.55, SD = 0.986), ‘Evidence is needed to inform programme planning and best practice in culture and sport’ (mean = 2.00, SD = 0.853) and ‘Evidence should help to understand the experiences of those who take part in culture and sport’ (mean = 2.70, SD = 0.853). Lower ranked were ‘Evidence should identify potentially negative wellbeing impacts of culture and sport’ and ‘Evaluation should be as independent as possible’ (mean = 4.70, SD = 0.687).

Respondents were asked, ‘what forms of evidence and evaluation are most needed?’ The higher ranking statements (Supplementary Table 7) were the following: ‘Evaluation should identify what doesn’t work as well as what works’ (mean = 1.78, SD = 1.330), ‘There is a need for longitudinal evidence in culture and sport’ (mean = 2.08, SD = 0.888) and ‘We need evidence of how things work not just outcomes measurement’ (mean = 3.08, SD = 0.829). Lower ranked were ‘Evaluation should be embedded in practice and should not disrupt service delivery’ (mean = 3.90, SD = 0.841) and ‘Case studies and stories are needed in order to explain the wellbeing benefits of participation in culture and sport to participants and funders’ (mean = 4.18, SD = 1.279).

Respondents were asked, ‘How should the review respond to the state of the current evidence base in culture and sport?’ (Supplementary Table 8). The highest ranked statements were ‘The review should encompass evidence produced by a diverse group of stakeholders including professionals, practitioners and service delivery organisations’ (mean = 1.25, SD = 0.588), ‘We should prioritise helping to develop clear quality criteria that can be used in future evaluation in culture and sport’ (mean = 2.30, SD = 0.791) and ‘The review should help to develop theories of change based approaches to wellbeing evaluation’ (mean = 3.05, SD = 0.876). Lower ranked were as follows: ‘The review needs to recognise that the current evidence base for wellbeing in culture and sport is weak’ (mean = 3.90, SD = 0.810) and ‘We need to prioritise collating and evaluating published literature in culture and sport’ (mean = 4.50, SD = 1.038).

Participants were asked, ‘To what extent should a hierarchy of evidence inform the culture and sport evidence review?’ (Supplementary Table 9). Highest ranked statements were the following: ‘The review should include rigorous qualitative evidence in culture and sport’ (mean = 1.40, SD = 0.900), ‘The review should include examples or approaches that are relevant to small organisations, e.g. case studies’ (mean = 2.15, SD = 0.622) and ‘The review should include unpublished (grey) literature’ (mean = 3.14, SD = 0.770). Lower ranked were ‘The review should prioritise finding robust quantitative research, including RCTs, in culture and sport’ (mean = 3.78, SD = 1.025) and ‘The review should include anecdotal evidence and testimonials’ (mean = 4.53, SD = 1.012).

Participants were asked, ‘How should the review approach quality assessment?’ (Supplementary Table 10). Highest ranked statements were the following: ‘Quality assessment in evidence should include the extent to which evaluation has been informed by the views of those who take part in culture and sport’ (mean = 1.25, SD = 0.630), ‘The review should adopt a clear step wise progression from lower quality evidence to gold standard’ (mean = 2.30, SD = 0.791) and ‘Applying health based hierarchies of evidence will overlook good evidence in culture and sport’ (mean = 2.88, SD = 0.853). Lower ranked were ‘Quality assessment in evidence should include the extent to which the evaluation has been informed by stakeholder views’ (mean = 3.95, SD = 0.597) and ‘The review should only include evidence that meets accepted quality criteria in quantitative and qualitative research’ (mean = 3.95, SD = 0.925).

Participants elaborated on dimensions of wellbeing relevant to culture and sport. These were divided into personal dimensions, sector-specific dimensions and social dimensions (Supplementary Table 11). The results regarding personal dimensions of wellbeing reveal priorities of ‘confidence and self-esteem’, ‘happiness’ and ‘meaning and purpose’ (respective means = 1.28, 2.48, 2.83, SDs = 0.679, 0.1062, 0.931). ‘Reduced anxiety’ and ‘optimism’ were ranked lower (respective means = 3.83, 4.60, SDs = 0.712, 0.778). Regarding sector-specific dimensions, priorities are ‘coping and resilience’ (mean = 1.40, SD = 0.672), ‘capability and achievement’ (mean = 2.25, SD = 0.670) and ‘personal identity’ (mean = 2.65, SD = 0.893). ‘Sporting or creative skills and expression’ and ‘life skills such as employability’ were ranked lower (respective means = 4.00, 4.70, SDs = 0.716, 0.853). Regarding social dimensions of wellbeing, highest ranked were ‘belonging and social identity’ (mean = 1.18, SD = 0.675), ‘sociability and new connections’ (mean = 2.48, SD = 0.905) and ‘bonding and social capital’ (mean = 3.23, SD = 0.733). After this came ‘reducing social inequalities’ (mean = 3.55, SD = 1.037) and ‘reciprocity and giving to others’ (mean = 4.58, SD = 0.874).

Participants were asked, ‘What populations are effective wellbeing interventions likely to be working with?’ (Supplementary Table 12). Higher priority are ‘General population: open access community-based culture and sport’ (mean = 1.25, SD = 0.630), ‘Specific, targeted populations (e.g. age, gender, ethnicity, low income, disability)’ (mean = 1.93, SD = 0.350) and ‘People who have been identified as having a specific health condition’ (mean 3.00, SD = 0.641). Lower ranked were ‘People in targeted geographical areas’ and ‘People who are members of cultural and sporting interest groups’ (respective means = 3.90, 4.93, SDs = 0.496, 0.350).

Participants were asked, ‘What settings are most likely to be delivering effective wellbeing interventions?’ (Supplementary Table 13). Priorities were ‘Community-based culture, sport and leisure’ (mean = 1.18, SD = 0.501), ‘School based arts and culture and sport’ (mean = 2.12, SD = 0.501) and ‘NHS/social care, statutory & third sector’ (mean = 2.98, SD = 0.530). Lower priority were ‘School based sport’ and ‘Culture and sport in commercial organisations’ (respective means = 4.03, 4.65, SDs = 0.486, 0.893).

Finally, participants were asked, ‘What culture and sport interventions are likely to have the strongest impact on wellbeing?’ (Supplementary Table 14). Highest priorities were ‘Group based interventions led by a volunteer or peer’ (mean = 1.25, SD = 0.588), ‘Individual activity that can be done alone’ (mean = 2.28, SD = 0.506) and ‘Group based interventions led by a professional’ (mean = 2.60, SD = 0.788). Lower ranked were ‘Non-active sport and culture (e.g. TV)’ and ‘Taking part in “elite” culture and sport’ (respective means = 4.23, 4.65, SDs = 0.698, 0.622).

In terms of settings, participants’ responses suggest that evidence for wellbeing impacts of culture and sport will be found across communities, schools, National Health Service (NHS)/social care, statutory and third sector as well as commercial organisations. Finally, in terms of delivery models, the approaches include group-based interventions led by volunteers, peers and professionals, individual activity that can be done alone, non-active sport and culture and taking part in ‘elite’ culture and sport.

## Discussion

The outcomes of a DELPHI process can be influenced by the placement of the threshold of consensus. A high threshold will produce fewer consensus statements, while a low threshold may mask important disagreements. We have not sought to generate definitive answers using the DELPHI in this study or impose a rigid threshold of consensus. To a degree, all the statements are important to stakeholders as they are drawn from an extensive consultation process in which a range of views were expressed. Instead, we have sought to illustrate broad and sometimes nuanced priorities that will inform the scope and parameters of a 3-year evidence review programme examining the impact of culture and sport on wellbeing.

The interpretation of the quantitative results is informed by the qualitative analysis which revealed four broad themes including definitions and dimensions of wellbeing, the purpose of evaluation, hierarchies of evidence, and culture, health and wellbeing populations, settings and interventions.

With regard to definitions and dimensions of wellbeing, the results show that there is broad support for the adoption of a common definition of wellbeing on which to base evaluation and research. The value of the ONS4 is recognised, but other dimensions are relevant to the culture and sport sectors, such as personal dimensions of wellbeing; for example, confidence and self-esteem, happiness, and meaning and purpose, sector-specific dimensions such as coping and resilience, capability and achievement and personal identity, and social dimensions, including belonging and social identity, sociability and new connections, and bonding and social capital. These dimensions may not represent a complete fit with the ONS4, but they reflect practice in the culture and sport sectors and therefore need to be taken into account in evidence review.

With regard to the purpose of evaluation, there is strong acceptance of the principle that evidence is needed to secure and maintain funding for culture and sport and that its evaluation is needed to inform programme planning and best practice in culture and sport. There is also general support for the principle that evaluation should identify what does not work as well as what works.

It is with regard to hierarchies of evidence that some interesting results have emerged. Discussions about hierarchies of evidence in workshops, and the resulting statements and priorities, reflect the particularities of the sector and may appear to be at odds with conventional wisdom in public health. During workshops, participants devoted considerable energy to discussing the current state of evidence in culture and sport. They gave strong support to the idea of a collaborative programme of evidence generation that engages diverse groups of stakeholders. They also recognised the need for the development of clear quality criteria that are relevant to assessment of evidence in culture and sport. However, participants ranked rigorous qualitative evidence, case studies and grey literature more highly than they did experimental methods and randomised controlled trials (RCTs). This reflects the state of the evidence in culture and sport, where most projects are evaluated using mixed methods and many evaluations are unpublished reports. Methodologies such as RCTs are only beginning to be applied and have often been viewed as challenging and inappropriate in complex and sensitive contexts.^1,25^ Sector particularities may have also influenced participants’ statements in relation to quality assessment, where there is an emphasis on consultation and participant engagement as well as recognition of the need for progression from lower quality evidence to gold standard. Thereseems to be some disagreement about what constitutes low quality evidence as well as scepticism towards the application of health-based hierarchies and fear that this will overlook evidence that is valued by stakeholders in culture and sport.

Regarding populations, settings and interventions, a number of priorities emerged. Priority populations are community-based culture and sport populations as well as specific populations targeted because of demographic and socioeconomic characteristics or existing health conditions. Priority-settings are communities, schools and health care rather than commercial organisations. Priority interventions are group based and individual, but not necessarily passive forms of engagement or taking part in ‘elite’ culture and sport. The results also indicate the populations, settings and intervention models which might reveal best evidence.

Regardless of whether consensus is reached, the DELPHI process records all views and therefore allows minority viewpoints to be clearly identified. These may be just as important to policy makers, stakeholders and researchers as majority views. Our study shows that there is broad agreement across the three groups of stakeholders across these key principles and parameters. There were some differences in the priorities of three groupings included in the study: SDOs seem to favour inductive, qualitative approaches to evaluation that do not disrupt service delivery, and are also more ready than others to recognise the value of anecdotal evidence and testimonials. Apart from this, there were no significant differences between the groups. These sub-group differences at DELPHI 1 did not prevent the group from reaching an overall consensus at DELPHI 2.

There are few areas of strong disagreement in these DELPHI results; however, the nuanced views, particularly in relation to the conduct of evaluation as well as hierarchies of evidence and quality assessment, reveal that SDOs may have slightly different agendas when it comes to evaluation and evidence than other groups. This may reflect the relatively early stage of development of the evidence base in this field and indicates a need for ongoing consultation to ensure that nobody is left behind when it comes to development and application of evaluation methodologies.

## Strengths and Limitations of The Study

A strength of the study is the response rate of 70.02% from a purposeful sample of participants selected on the basis of their organisational role and involvement across a range of CSW contexts. However, this was not a comprehensive survey of a representative sample of organisations and stakeholders involved in CSW. Time and resources prohibited this, but the Delphi technique has proved useful for including a focused sample of participants from diverse backgrounds in deliberation and consensus development. It has the advantage of being democratic in that it allows all participants an equal chance of influencing the results.

It could be said that the presentation of statements in rank order at DELPHI 2 was leading. However, DELPHI 2 was the culmination of 6 months of collaborative development in face-to-face workshops, interviews, observations and online communication with stakeholders, many of whom the research team have known and been collaborating with for several years. We would not have expected substantial reworking of priorities at this stage. In order to distinguish higher and lower priorities, we have applied a basic threshold of consensus, identifying the top three statements as higher priority. However, we recognise that this is somewhat arbitrary and that all the statements were of value to stakeholders.

## Conclusion

The DELPHI process, in combination with extensive face-to-face deliberation, has enabled stakeholders to engage in complex discussion and express nuanced priorities while also allowing a diverse group to come to an overall consensus and agree priorities. We have demonstrated the importance of consultation to ensure that professionals, practitioners and service users can contribute to evaluation and evidence review. The degree of consensus reached suggests that the resulting statement can inform the development of a broad strategy to assess the effect of policies and initiatives on wellbeing with which all the groups can agree.

The exercise revealed strong support across stakeholders for a common definition of wellbeing on which to base research and evidence, provided that this can encompass dimensions that are important in culture and sport, such as confidence and self-esteem, as well as social dimensions of wellbeing. The exercise also revealed strong support for evidence generation, to show wellbeing outcomes of culture and sport, to help to secure funding and therefore contribute to sustainability, to inform programme planning, and to understand the experiences of those who take part in culture and sport. Furthermore, the data showed clear support across stakeholder groups for the use of quality criteria to assess evaluation. Stakeholders’ aspirations to go beyond the ONS4 and focus on the importance of context may be revealing a preference for experiential measures of SWB in culture and sport. Our project will explore how people feel while they are engaged in experiences of culture and sport as well as identifying how culture and sport impact on SWB overall.

While there is support for quality standards in evidence review, the ambivalence that was revealed surrounding hierarchies of evidence, together with support for forms of evidence typically seen as lower quality, is perhaps a reflection of the relatively early stage of development of evidence generation for wellbeing outcomes in culture and sport.

The process has highlighted the dimensions of wellbeing that are important to the sector, as well as the populations, settings and types of intervention that it is important to focus on. Together with the analysis of the qualitative data, it has also helped to establish the topics and the parameters for the evidence reviews. Over the next 3 years, the research team will undertake reviews and secondary analysis of wellbeing outcomes of music and singing of adults (18+ years); sport and dance for adolescents and young people (14–25 years); visual arts for adults (18+ years) with a mental health condition; and sport and recreation across the family lifecourse. A cross-evidence review programme will examine wellbeing outcomes of co-produced culture and sport, community, and work and learning interventions. Each review will examine the processes by which wellbeing outcomes are achieved and will include analysis of all protected characteristics (age, sex, gender reassignment, sexual orientation, disability, race, religion, pregnancy/maternity, marriage/civil partnerships)^32^ and income and/or socioeconomic status. Hence, while we have not sought to generate definitive answers from this DELPHI, the process has helped to clarify the aims and scope of the CSW evidence review programme. This is of value because the systematic study of wellbeing in culture and sport has been prioritised by programmes such as the What Works Programme in order to strengthen policy making, commissioning and funding of projects and programmes.

## Supplementary Material

Supplementary material
