# Supplementary material for: What works for wellbeing in culture and sport? Report of a DELPHI process to support coproduction and establish principles and parameters of an evidence review
Source: Perspect Public Health. 2016 Oct 28;137(5):281–8. doi: 10.1177/1757913916674038 (PMC5582641; doi:10.1177/1757913916674038)
Supplement: Supplementary material [file PPH674038_Supplementary.pdf]

What Works for Wellbeing in Culture and Sport? Report of a DELPHI process to support coproduction and establish principles and parameters of an evidence review.

Table 1: Delphi 1 results

	N	Mean	Std. Deviation
**1. What is meant by wellbeing in the context of the culture and sport evidence review?**			
1.1 We need a common definition of wellbeing on which to base evaluation and research	38	1.84	1.079
1.2 We need to avoid reductionist, over-simplistic approaches in order to capture complex dimensions of wellbeing	38	2.26	1.201
1.3 We need to adopt an inductive approach that assesses wellbeing in each specific context	38	2.32	.989
1.4 The ONS four dimensions of wellbeing (life satisfaction, worthwhileness, happiness and anxiety) are important to the sector.	37	2.11	1.022
1.5 Not all the ONS dimensions of wellbeing are relevant to the sector	37	3.49	1.261
1.6 We need to include a range of wellbeing dimensions into evidence reviews in addition to the ONS4.	37	1.97	1.118
**2. What is the purpose of evidence in culture and sport?**			
2.1 Evidence is needed to secure and maintain funding for culture and sport	38	1.37	.786
2.2 Evaluation should be distinct from advocacy in culture and sport	37	2.16	1.214
2.3 Evidence is needed to inform programme planning and best practice in culture and sport	38	1.53	1.033
2.4 Evidence should help to understand the experiences of those who take part in culture and sport	38	1.55	.978
2.5 Evidence should identify potentially negative wellbeing impacts of culture and sport	38	1.61	1.001
2.6 Evaluation should be as independent as possible	38	1.87	1.070
**3. What forms of evidence and evaluation are most needed?**			
3.1 We need to prioritise evidence for outcome measures of wellbeing	38	2.21	.905
3.2 We need evidence of how things work not just outcomes measurement	38	1.63	1.025
3.3 Case studies and stories are needed in order to explain the wellbeing benefits of participation in culture and sport to participants and funders	38	1.76	1.149
3.4 There is a need for longitudinal evidence in culture	37	1.54	.931

and sport			
3.5 We need evidence of what causes wellbeing in culture and sport	38	1.79	1.094
3.6 Evaluation should be embedded in practice and should not disrupt service delivery	37	1.73	1.071
3.7 Evidence should be tailored to the requirements of commissioners and funders	36	2.61	1.103
3.8 Evaluation should identify what doesn't work as well as what works	38	1.34	.878
**4. What forms of evidence and evaluation are most needed?**			
4.1 The review needs to recognise that the current evidence base for wellbeing in culture and sport is weak	38	2.00	1.139
4.2 We need to prioritise collating and evaluating published literature in culture and sport	38	2.34	.938
4.3 We need to prioritise collating and evaluating grey (unpublished) literature in culture and sport	38	2.61	1.079
4.4 We should prioritise helping to develop clear quality criteria that can be used in future evaluation in culture and sport	38	1.89	1.134
4.5 The review should help to develop theories of change based approaches to wellbeing evaluation	38	1.97	1.052
4.6 The review should encompass evidence produced by a diverse group of stakeholders including professionals, practitioners and service delivery organisations	38	1.45	.978
**5. To what extent should a hierarchy of evidence inform the culture and sport evidence review?**			
5.1 The review should prioritise finding robust quantitative research, including RCTs, in culture and sport	38	2.16	1.079
5.2 The review should include rigorous qualitative evidence in culture and support	38	1.50	.862
5.3 The review should only include evidence that has been peer reviewed	38	3.71	1.011
5.4 The review should include unpublished (grey) literature	37	1.95	.848
5.5 The review should include anecdotal evidence and testimonials.	38	2.58	1.030
5.6 The review should include examples or approaches that are relevant to small organisations, e.g. case studies.	38	1.66	.938
5.7 The best evidence for culture and sport is generated by the participants themselves	38	2.63	1.172
**6. How should the review approach quality assessment?**			
6.1 The review should adopt a clear step wise	36	2.08	1.025

progression from lower quality evidence to gold standard			
6.2 Quality assessment in evidence should include the extent to which the evaluation has been informed by stakeholder views	37	2.38	1.010
6.3 Quality assessment in evidence should include the extent to which evaluation has been informed by the views of those who take part in culture and sport	38	2.03	.972
6.4 The review should only include evidence that meets accepted quality criteria in quantitative and qualitative research	38	2.68	1.141
6.5 Applying health based hierarchies of evidence will overlook good evidence in culture and sport	36	2.14	1.125
6.6 Applying health based hierarchies of evidence will demoralise those involved in the field and crush good practice	35	2.71	1.178
**7. What key dimensions of wellbeing should be included in the review?**			
7a) Personal dimensions of wellbeing			
7a.1 Happiness	38	1.82	.955
7a.2 Confidence and self-esteem	38	1.74	1.057
7a.3 Optimism	38	2.58	1.200
7a.4 Reduced anxiety	38	2.24	1.261
7a.5 Meaning and purpose	38	2.05	1.229
7b) Culture/sport based dimensions of wellbeing			
7b.1 Capability and achievement	38	1.97	.915
7b.2 Personal identity	38	2.08	1.148
7b.3 Life skills such as employability	38	2.53	1.350
7b.4 Sporting or creative skills and expression	38	2.50	1.289
7b.5 Coping and resilience	38	1.89	1.110
7c) Social dimensions of wellbeing			
7c.1 Belonging and social identity	38	1.61	.887
7c.2 Bonding and social capital	38	2.08	1.100
7c.3 Sociability and new connections	38	1.92	.941
7c.4 Reciprocity and giving to others	38	2.50	1.371
7c.5 Reducing social inequalities	38	2.13	1.298
**8. What populations are effective wellbeing interventions likely to be working with?**			
8.1 General population: open access community based culture and sport.	38	1.66	1.021
8.2 People who are members of cultural and sporting interest groups	38	2.39	1.462
8.3 Specific, targeted populations (e.g. age, gender, ethnicity, low income, disability)	37	1.65	1.006
8.4 People who have been identified as having a specific health condition	38	2.21	1.166
8.5 People in targeted geographical areas	38	2.24	1.344

**9. What settings are most likely to be delivering effective wellbeing interventions?**			
9.1 School based arts and culture and sport	38	1.92	1.024
9.2 School based sport	36	2.19	1.064
9.3 Community based culture, sport and leisure	38	1.45	.828
9.4 Specialist 'elite' culture and sport settings	36	3.42	1.339
9.5 NHS/social care, statutory & third sector.	37	2.19	1.198
9.6 Culture and sport in commercial organisations.	37	3.08	1.278
**10. What culture and sport interventions are likely to have the strongest impact on wellbeing?**			
10.1 Group based interventions led by a professional	38	1.76	1.076
10.2 Group based interventions led by a volunteer or peer	38	1.63	1.051
10.3 Individual activity that can be done alone	38	1.71	.898
10.4 Non active sport and culture (e.g. TV)	38	3.13	1.436
10.5 Taking part in 'elite' culture and sport	38	3.24	1.515

Table 2. Statements ranked highly by service delivery organisations, DELPHI 1.

	Service Delivery Organisations			Overall rankings			p-value and significance
1. What is meant by wellbeing in the context of the culture and sport evidence review?	N	Mean	Std. Dev	N	Mean	Std. Dev	-
We need to adopt an inductive approach that assesses wellbeing in each specific context	8	1.50 (2nd)	.926	38	2.32 (5th)	.989	0.0369 * Significant
3. What forms of evidence and evaluation are most needed?							
Evaluation should be embedded in practice and should not disrupt service delivery	7	1.43 (2nd)	1.134	37	1.73 (4th)	1.071	0.5041 ns Not significant
5. To what extent should a hierarchy of evidence inform the culture and sport evidence review?							
The review should include anecdotal evidence and testimonials.	8	1.38 (1st)	1.061	38	2.58 (5th)	1.030	0.0047 ** Very significant

Table 3. Statements ranked highly by commissioners and managers, DELPHI 1.

Statement	Commissioners	Overall rankings	p-value and significance

4. What forms of evidence and evaluation are most needed?	N	Mean	Std. Dev	N	Mean	Std. Dev	
Case studies and stories are needed in order to explain the wellbeing benefits of participation in culture and sport to participants and funders	9	1.56 (1st)	1.333	38	1.76 (5th)	1.149	0.6378 ns Not significant

Table 4. Statements ranked highly by scholars and policy makers, DELPHI 1.

Statement	Scholars			Policy Makers			Overall rankings			p-value and significance	
2. What is the purpose of evidence in culture and sport?	N	Mean	Std. Dev	N	Mean	Std. Dev	N	Mean	Std. Dev	Scholars vs overall	Policy Makers vs overall
Evaluation should be as independent as possible	10	1.40 (2nd)	.699	11	1.55 3rd	.688	38	1.87 5th	1.070	0.1961 ns Not significant	0.3560 ns Not significant

**P value**	**Wording**	**Summary**
< 0.0001	Extremely significant	****
0.0001 to 0.001	Extremely significant	***
0.001 to 0.01	Very significant	**
0.01 to 0.05	Significant	*
≥ 0.05	Not significant	ns

Table 5. DELPHI 2 top five statements for 'What is meant by wellbeing in the context of the culture and sport evidence review? in rank order: means and standard deviations (lowest mean is highest rank)

**What is meant by wellbeing in the context of the culture and sport evidence review?**	N	Mean	Std. Deviation

1. We need a common definition of wellbeing on which to base evaluation and research	40	1.50	1.109
2. We need to include a range of wellbeing dimensions into evidence reviews in addition to the ONS4.	40	2.20	.648
3. The ONS four dimensions of wellbeing (life satisfaction, worthwhileness, happiness and anxiety) are important to the sector.	40	3.08	.694
4. We need to avoid reductionist, over-simplistic approaches in order to capture complex dimensions of wellbeing	40	3.73	.960
5. We need to adopt an inductive approach that assesses wellbeing in each specific context	40	4.48	1.198

Table 6. DELPHI 2 top five statements for 'What is the purpose of evidence in culture and sport?' in rank order: means and standard deviations (lowest mean is highest rank).

**2. What is the purpose of evidence in culture and sport?**	N	Mean	Std. Deviation
1. Evidence is needed to secure and maintain funding for culture and sport	40	1.55	.986
2. Evidence is needed to inform programme planning and best practice in culture and sport	40	2.00	.716
3. Evidence should help to understand the experiences of those who take part in culture and sport	40	2.70	.853
4. Evidence should identify potentially negative wellbeing impacts of culture and sport	40	4.05	.450
5. Evaluation should be as independent as possible	40	4.70	.687

Table 7. DELPHI 2 top five statements for 'What forms of evidence and evaluation are most needed?' in rank order: means and standard deviations (lowest mean is highest rank).

**3. What forms of evidence and evaluation are most needed?**	N	Mean	Std. Deviation
1. Evaluation should identify what doesn't work as well as what works	40	1.78	1.330
2. There is a need for longitudinal evidence in culture and sport	40	2.08	.888
3. We need evidence of how things work not just outcomes measurement	40	3.08	.829
4. Evaluation should be embedded in practice and should not disrupt service delivery	40	3.90	.841
5. Case studies and stories are needed in order to explain the wellbeing benefits of participation in culture and sport to participants and funders	40	4.18	1.279

Table 8. DELPHI 2 top five statements for 'How should the review respond to the state of the current evidence base in culture and sport?' in rank order: means and standard deviations (lowest mean is highest rank).

**4. How should the review respond to the state of the current evidence base in culture and sport?**	N	Mean	Std. Deviation
1. The review should encompass evidence produced by a diverse group of stakeholders including professionals, practitioners and service delivery organisations	40	1.25	.588
2. We should prioritise helping to develop clear quality criteria that can be used in future evaluation in culture and sport	40	2.30	.791
3. The review should help to develop theories of change based approaches to wellbeing evaluation	40	3.05	.876
4. The review needs to recognise that the current evidence base for wellbeing in culture and sport is weak	40	3.90	.810
5. We need to prioritise collating and evaluating published literature in culture and sport	40	4.50	1.038

Table 9. DELPHI 2 top five statements for 'To what extent should a hierarchy of evidence inform the culture and sport evidence review?' in rank order: means and standard deviations (lowest mean is highest rank).

**5. To what extent should a hierarchy of evidence inform the culture and sport evidence review?**	N	Mean	Std. Deviation
1. The review should include rigorous qualitative evidence in culture and support	40	1.40	.900
2. The review should include examples or approaches that are relevant to small organisations, e.g. case studies.	40	2.15	.622
3. The review should include unpublished (grey) literature	40	3.15	.770
4. The review should prioritise finding robust quantitative research, including RCTs, in culture and sport	40	3.78	1.025
5. The review should include anecdotal evidence and testimonials.	40	4.53	1.012

Table 10. DELPHI 2 top five statements for 'How should the review approach quality assessment?' in rank order: means and standard deviations (lowest mean is highest rank).

**6. How should the review approach quality assessment?**	N	Mean	Std. Deviation
1. Quality assessment in evidence should include the extent to which evaluation has been informed by the views of those who take part in culture and sport	40	1.25	.630
2. The review should adopt a clear step wise progression from lower quality evidence to gold standard	40	2.30	.791

3. Applying health based hierarchies of evidence will overlook good evidence in culture and sport	40	2.88	.853
4. Quality assessment in evidence should include the extent to which the evaluation has been informed by stakeholder views	40	3.95	.597
5. The review should only include evidence that meets accepted quality criteria in quantitative and qualitative research	40	4.63	.925

Table 11. DELPHI 2 top five statements for 'What key dimensions of wellbeing should be included in the review?' in rank order: means and standard deviations (lowest mean is highest rank).

**7. What key dimensions of wellbeing should be included in the review?**	N	Mean	Std. Deviation
Personal dimensions of wellbeing			
1. Confidence and self-esteem	40	1.28	.679
2. Happiness	40	2.48	1.062
3. Meaning and purpose	40	2.83	.931
4. Reduced anxiety	40	3.83	.712
5. Optimism	40	4.60	.778
Culture/sport based dimensions of wellbeing			
1. Coping and resilience	40	1.40	.672
2. Capability and achievement	40	2.25	.670
3. Personal identity	40	2.65	.893
4. Sporting or creative skills and expression	40	4.00	.716
5. Life skills such as employability	40	4.70	.853
Social dimensions of wellbeing			
1. Belonging and social identity	40	1.18	.675
2. Sociability and new connections	40	2.48	.905
3. Bonding and social capital	40	3.23	.733
4. Reducing social inequalities	40	3.55	1.037
5. Reciprocity and giving to others	40	4.58	.874

Table 12. DELPHI 2 top five statements for 'What populations are effective wellbeing interventions likely to be working with?' in rank order: means and standard deviations (lowest mean is highest rank).

**8. What populations are effective wellbeing interventions likely to be working with?**	N	Mean	Std. Deviation
8.1 General population: open access community-based culture and sport.	40	1.25	.630
8.2 Specific, targeted populations (e.g. age, gender, ethnicity, low	40	1.93	.350

income, disability)			
8.3 People who have been identified as having a specific health condition	40	3.00	.641
8.4 People in targeted geographical areas	40	3.90	.496
8.5 People who are members of cultural and sporting interest groups	40	4.93	.350

Table 13. DELPHI 2 top five statements for 'What settings are most likely to be delivering effective wellbeing interventions?' in rank order: means and standard deviations (lowest mean is highest rank).

9. **What settings are most likely to be delivering effective wellbeing interventions?**	N	Mean	Std. Deviation
9.1 Community-based culture, sport and leisure	40	1.18	.501
9.2 School based arts and culture and sport	40	2.13	.723
9.3 NHS/social care, statutory & third sector.	40	2.98	.530
9.4 School based sport	39	4.03	.486
9.5 Culture and sport in commercial organisations.	40	4.65	.893

Table 14. DELPHI 2 top five statements for 'What settings are most likely to be delivering effective wellbeing interventions?' in rank order: means and standard deviations (lowest mean is highest rank).

10. **What culture and sport interventions are likely to have the strongest impact on wellbeing?**	N	Mean	Std. Deviation
10.1 Group based interventions led by a volunteer or peer	40	1.25	.588
10.2 Individual activity that can be done alone	40	2.28	.506
10.3 Group based interventions led by a professional	40	2.60	.778
10.4 Non active sport and culture (e.g. TV)	40	4.23	.698
10.5 Taking part in 'elite' culture and sport	40	4.65	.622
